# Increase in Motor Vehicle Crash Severity: An Unforeseen Consequence of COVID-19

**DOI:** 10.1177/00031348211047466

**Published:** 2021-10-13

**Authors:** Elinore J. Kaufman, Daniel Holena, George Koenig, Niels D. Martin, George O. Maish, Benjamin J. Moran, Asanthi Ratnasekera, Stanislaw P. Stawicki, Marie Timinski, Joshua Brown

**Affiliations:** 1 Division of Trauma, Surgical Critical Care, and Emergency Surgery, 6572 University of Pennsylvania, Philadelphia, PA, USA; 2 6559 Thomas Jefferson University, Philadelphia, PA, USA; 3 Division of Trauma and Acute Care Surgery, Penn Medicine Lancaster General Health, Lancaster, PA, USA; 4 6566 Einstein Medical Center, Philadelphia, PA, USA; 5 Department of Surgery, 22901 Crozer Chester Medical Center, Upland, PA, USA; 6 Department of Research & Innovation, St. Luke’s University Health Network, Bethlehem, PA, USA; 7 5309 Geisinger Wyoming Valley, Wilkes Barre, PA, USA; 8 Division of Trauma and General Surgery, Department of Surgery, 6595 University of Pittsburgh Medical Center, Pittsburgh, PA, USA

**Keywords:** trauma, public health, geospatial analysis, epidemiology, motor vehicle crashes

## Abstract

**Introduction:**

The 2019 coronavirus (COVID-19) pandemic led to stay-at-home (SAH) orders in Pennsylvania targeted at reducing viral transmission. Limitations in population mobility under SAH have been associated with decreased motor vehicle collisions (MVC) and related injuries, but the impact of these measures on severity of injury remains unknown. The goal of this study is to measure the incidence, severity, and outcomes of MVC-related injuries associated with SAH in Pennsylvania.

**Materials & Methods:**

We conducted a retrospective geospatial analysis of MVCs during the early COVID-19 pandemic using a state-wide trauma registry. We compared characteristics of patients with MVC-related injuries admitted to Pennsylvania trauma centers during SAH measures (March 21-July 31, 2020) with those from the corresponding periods in 2018 and 2019. We also compared incidence of MVCs for each zip code tabulation area (ZCTA) in Pennsylvania for the same time periods using geospatial mapping.

**Results:**

Of 15,550 trauma patients treated during the SAH measures, 3486 (22.4%) resulted from MVCs. Compared to preceding years, MVC incidence decreased 10% under SAH measures with no change in mortality rate. However, in ZCTA where MVC incidence decreased, there was a 16% increase in MVC injury severity.

**Conclusions:**

Stay-at-home orders issued in response to the COVID-19 pandemic in Pennsylvania were associated with significant changes in MVC incidence and severity. Identifying such changes may inform resource allocation decisions during future pandemics or SAH events.

## Introduction

In response to the coronavirus disease 2019 (COVID-19) pandemic, the Pennsylvania stay-at-home (SAH) order was in effect between March 16, 2020 and June 4, 2020, with various restrictions remaining in place thereafter.^1-3^ Changes in population mobility under SAH measures have been associated with shifts in trauma volume and complexity,^4,5^ including reports of fewer traffic-related injuries.^6,7^ There are no reports evaluating injury severity characteristics among traffic collision victims in the setting of lower mobility and decreased trauma admissions. Identifying changes in injury patterns and trauma admission volumes is important to health system preparation and resource allocation during resurgences and future pandemics. To examine how the geospatial patterns of injury in Pennsylvania were affected by COVID-19 public health policies, we focused on motor vehicle collision (MVC) injury events. Motor vehicle collisions are influenced by numerous geographic and social factors,^8^ and care for MVC patients mobilizes the full range of prehospital and trauma center resources.^9^ The objective of this study was to measure the incidence, severity, and outcomes of MVC-related injuries after implementation of SAH in Pennsylvania.

## Methods

### Data Source and Population

We used the Pennsylvania Trauma Outcomes Study (PTOS), a state-wide trauma registry maintained by the Pennsylvania Trauma Systems Foundation (PTSF, Mechanicsburg, Pa) as our data source. Patients with primary burn injury mechanism were excluded. We defined the starting point for the COVID-19 era (C19E) as March 21, 2020, corresponding to Pennsylvania’s implementation of non-essential business closures as part of SAH measures.^10^ To construct temporal control groups, our primary sample included patients involved in MVCs and admitted between March 21 and July 31, 2020 and our control groups were derived using the corresponding time periods in 2018 and 2019.

### Outcomes

The primary outcome was incidence of MVCs in each time period. Secondary outcomes included injury severity, all-cause mortality, all-type morbidity, hospital length of stay, ventilator days, and intensive care unit length of stay (ICULOS).

### Analysis

Descriptive analyses were performed using measures of central tendencies, chi-squared testing for categorical outcomes, and the Kruskal-Wallis test for continuous, non-normally distributed outcome variables using Stata Version 16 (StataCorp, College Station, TX). A *P* value of <.05 was considered significant.

### Geospatial Analysis

We calculated the rate of MVCs per 10,000 population for each zip code tabulation area (ZCTA)^11^ in Pennsylvania during the C19E and in the corresponding time periods for 2018 and 2019.^12^ Zip code tabulation areas without a recorded MVC-related patient injury in either the pre-C19E or C19E periods were excluded. Median injury severity scores (mISS) were calculated in MVC patients for the 2 years preceding the C19E and during the C19E at the ZCTA level. We mapped the percent change in MVC rate and mISS for each ZCTA and categorized ZCTAs as decreasing (percent change ≤−5%), no change (−5% to + 5%), or increasing (≥5%) for MVC rate and mISS. The resulting 9 combinations of “percent change” categories in MVC rate and mISS were converted into visualizations using bivariate choropleth mapping (ArcGIS v10.8, ESRI, Redlands, CA) to examine the relationship between change in MVC rate and mISS during the pre-C19E and C19E periods.

## Results

Of 15,550 trauma patients treated during the C19E, 3486 (22.4%) were injured in MVCs, compared to 3876/16,852 (23.0%) in 2018 and 3876/17,214 (22.5%) in 2019. Incidence of MVCs decreased by 10% during the C19E time period relative to both the 2018 and 2019 time periods. Patient demographics, physiology, comorbidities, and injury characteristics are shown in Table 1. Patients were younger during C19E (median age 39 in C19E vs. 46 in 2019 and 45 in 2018) with greater proportion of males. During C19E, more patients were black or Hispanic and fewer were Caucasian. Table 2 shows the similarity in outcomes for MVC-injured patients between time periods.

**Table 1.** Characteristics of Motor Vehicle Crash Patients Treated in 2018, 2019, and 2020, with 2020 representing the stay-at-home COVID-19 period in Pennsylvania.

	3/21-7/31/2018	3/21-7/31/2019	3/21-7/31/2020	*P* value
	N = 3876	N = 3876	N = 3486	
Male	2476 (63.9)	2491 (64.3)	2417 (69.3)	<0.001
Female	1400 (36.1)	1385 (35.7)	1069 (30.7)	
Age^a^	45 (27, 62)	46 (28, 62)	39 (25, 57)	<0.001
Race/ethnicity				<0.001
White	3086 (81.2)	3054 (80.6)	2564 (75.0)	
Black	423 (11.1)	458 (12.1)	558 (16.3)	
Hispanic/Latinx	187 (4.9)	202 (5.2)	241 (7.1)	
Asian	45 (1.2)	38 (0.8)	19 (0.6)	
Multiracial/other	62 (1.6)	46 (1.2)	38 (1.1)	
Insurance				<0.001
Medicare	301 (7.8)	338 (8.7)	222 (6.4)	
Medicaid	523 (13.5)	495 (12.8)	613 (17.6)	
Private	1026 (26.2)	1017 (26.2)	957 (27.5)	
Auto insurance or other	1872 (48.3)	1813 (46.8)	1461 (41.9)	
None or unknown	164 (4.2)	213 (5.5)	233 (6.7)	
Systolic blood pressure^a^	136 (121, 152)	137 (121, 152)	136 (120, 150)	0.049
Heart rate^a^	88 (76, 102)	88 (77, 102)	89 (77, 102)	0.33
GCS^a^	15 (15, 15)	15 (15, 15)	15 (15, 15)	0.24
Injury Severity Score^a^	9 (5, 15)	10 (5, 14)	10 (5, 14)	0.004
Maximum AIS^a^	3 (2, 3)	3 (2, 3)	3 (2, 3)	0.003
Body regions injured (AIS ≥2)				
Head	945 (24.4)	989 (25.5)	948 (27.2)	0.022
Face	409 (10.6)	378 (9.8)	381 (10.9)	0.24
Chest	1,531 (39.5)	1,542 (39.8)	1,404 (40.3)	0.79
Abdomen	500 (12.9)	516 (13.3)	441 (12.7)	0.69
Extremities	1897 (48.9)	1937 (50.0)	1860 (53.4)	<0.001
Comorbidities				
Heart disease	297 (7.7)	301 (7.8)	233 (6.7)	0.15
Lung disease	226 (5.8)	203 (5.2)	171 (4.9)	0.20
Liver disease	32 (0.8)	30 (0.8)	33 (1.0)	0.71
Cancer	23 (0.6)	27 (0.7)	23 (0.7)	0.85
Hypertension	1,113 (28.7)	1098 (28.3)	854 (24.5)	<0.001
Diabetes	481 (12.4)	487 (12.6)	349 (10.0)	0.001
Coagulopathy/anticoagulants/antiplatelet	639 (16.5)	660 (17.0)	458 (13.1)	<0.001
Dementia	21 (0.5)	35 (0.6)	24 (0.7)	0.71
Obesity	881 (22.7)	931 (24.0)	824 (23.6)	0.39
Dialysis	15 (0.4)	9 (0.2)	16 (0.5)	0.24

^a^Median (interquartile range). All others N (%).

**Table 2:** Outcomes of Pennsylvania Motor Vehicle Crash Patients.

	3/21-7/31/2018	3/21-7/31/2019	3/21-7/31/2020	*P* value
Died	164 (4.7)	147 (3.8)	165 (4.3)	0.15
Length of stay^a^	3 (2, 6)	3 (2, 6)	3 (2, 6)	0.038
ICU admission	1292 (37.1)	1524 (39.3)	1400 (36.1)	0.012
ICU length of stay (if admitted to ICU)^a^	2 (1,5)	2 (1,5)	2 (1,4)	0.20
Ventilator days (if intubated)^a^	2 (1,9)	2 (1,8)	2 (1,7)	0.60
Complications				
Any complication	276 (7.9)	361 (9.3)	239 (8.5)	0.10
ARDS	10 (0.3)	13 (0.3)	8 (0.2)	0.55
Pneumonia	66 (1.9)	72 (1.9)	56 (1.4)	0.25
DVT/PE	44 (1.3)	57 (1.5)	49 (1.3)	0.66
Wound or surgical site infection	9 (0.3)	14 (0.4)	19 (0.5)	0.26
Renal failure	15 (0.4)	14 (0.4)	26 (0.7)	0.12
Unplanned ICU admission	67 (1.9)	95 (2.5)	84 (2.2)	0.30
Discharge destination				<0.001
Home/routine	2739 (78.6)	2792 (72.0)	2,790 (72.0)	
Rehab/SNF/LTACH	577 (16.6)	928 (23.9)	915 (23.6)	

^a^Median (interquartile range). All others N (%). Abbreviations: ARDS, Acute Respiratory Distress Syndrome; DVT, Deep Vein Thrombosis; PE, Pulmonary Embolism; ICU, Intensive Care Unit; SNF, Skilled Nursing Facility; LTACH, Long-term Acute Care Hospital.

**Figure 1.**
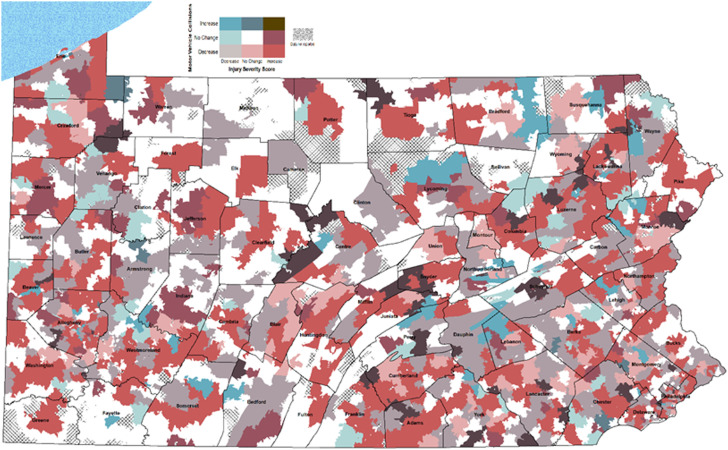
Bivariate choropleth map of change in motor vehicle collision rate per 10,000 population and change in median ISS at the zip code tabulation level from pre-quarantine periods to quarantine period in Pennsylvania. A decrease in the variable was designated if the percent change was < −5%, an increase designated if the percent change was >+5%, and no change if > −5% and < + 5% between the pre-quarantine periods and the quarantine period. Zip code tabulation areas with cross hatch did not have sufficient data for calculation.

Bivariate choropleth mapping shows significant geographic variation in the relationship between change in MVC rate and change in mISS among 1798 ZCTAs (Figure 1). Aside from “no change” and “no reported MVCs,” the most common pattern seen across the Commonwealth was a decrease in MVC rate coupled with an increase in mISS (15.5% of ZCTA). This was followed by areas with a decrease in both MVC rate and mISS (13.6% of ZCTA).

## Discussion

The total number of trauma admissions in Pennsylvania decreased during the C19E, and most geographic areas of the Commonwealth saw lower incidence of MVCs, although the overall proportion was relatively similar. However, in particular geographic locations, MVC severity increased significantly, with these local increases forming the most common pattern of change in MVCs.

Our results are consistent with those of Sutherland et al, who identified decreasing rates of MVCs during the COVID-19 pandemic.^13^ However, that study did not evaluate injury severity. In general, our observations are comparable to those from single-center urban-focused studies.^14^ The etiology of higher injury severity is unknown, but may include lower traffic volume allowing higher rates of speed,^15^ or riskier behavior among those driving. The latter may be associated with various demographic and behavioral characteristics of those actively on the road during the pandemic (eg, younger age, male predominance, and substance abuse).^16^ Our geographically focused results are generally consistent with national indicators showing a 24% increase in fatalities per mile driven, the highest annual increase in nearly 100 years.^17^

The sociology of pandemics creates secondary repercussions and an understanding of these downstream consequences should be part of any pandemic response. Globally reported trauma volumes during the peak of the COVID-19 pandemic declined between 20% and 84%, depending on locality and mechanism of injury.^18^ Although Pennsylvania also experienced this phenomenon, for large geographic regions in the state, the injury severity increased. Factors such as reduced traffic density leading to higher speeds of collisions may play a role, but require further examination. Increased anxiety and behavioral response has been well described during the COVID-19 pandemic,^16^ along with an increase in drug and alcohol use and abuse. These factors may augment severity in MVCs.^13,19^ Observed increases in U.S. traffic fatality rates contrast dramatically with a 12% decrease in German traffic deaths, after accounting for an approximately 11% decrease in traffic volume in that country.^17^ Our examination of state-level data during the pandemic SAH phase provides a unique snapshot representing a compilation of behavioral responses to the immense stress experienced by Pennsylvanians during this period of time. Findings of this study strongly suggest that although the absolute number of MVCs decreased as a result of the above, the severity of resultant injuries increased. Important public health considerations arise, including the need for early risk pattern recognition and appropriate preventive messaging to raise awareness of such patterns.

The current study’s findings underscore the utility of the geospatial methods to facilitate more granular explorations and to guide possible interventions. Limiting assessment to overall trends in MVCs and injury severity might suggest erroneous conclusions of minimal changes if opposite patterns are occurring in distinct geographic areas across the study region. Our collective experiences with trauma systems during the COVID-19 pandemic may be especially relevant during future SAH mandates and similar pandemic/outbreak-related restrictions. Based on our findings, combined with experiences from around the globe,^17^ it is reasonable to recommend that public education surrounding road safety should accompany future quarantine restrictions. Law enforcement and governmental agencies can use these data to support targeted MVC prevention efforts.

This study has several limitations. The PTOS data used in our study capture only patients presenting to trauma centers after MVC. Those treated at other hospitals or those who did not seek medical care are not included, nor are deaths that occurred on scene. Our data were limited to the early portion of the pandemic, and their durability remains to be determined. We chose a threshold of 5% change in MVC incidence or severity as clinically relevant, but other thresholds might yield different results. We used zip code of residence to attribute MVC to ZCTA, consistent with evidence that zip code of residence is an accurate proxy for injury location.^20^

## Conclusion

Overall, the absolute number of MVCs was lower during the pandemic SAH phase secondary to restricted mobility, but injury severity and fatality rates increased in particular geographic areas. The pandemic has strained public health strategies that have made roads, cars, and drivers so much safer over the last 3 decades, requiring increased attention to injury prevention and risk pattern recognition in future pandemics or resurgences.
